# Preparing for the bedside—optimizing a postpartum depression risk prediction model for clinical implementation in a health system

**DOI:** 10.1093/jamia/ocae056

**Published:** 2024-03-26

**Authors:** Yifan Liu, Rochelle Joly, Meghan Reading Turchioe, Natalie Benda, Alison Hermann, Ashley Beecy, Jyotishman Pathak, Yiye Zhang

**Affiliations:** Department of Population Health Sciences, Weill Cornell Medicine, New York, NY 10065, United States; Department of Obstetrics and Gynecology, Weill Cornell Medicine, New York, NY 10065, United States; Columbia University School of Nursing, New York, NY, United States; Columbia University School of Nursing, New York, NY, United States; Department of Psychiatry, Weill Cornell Medicine, New York, NY 10065, United States; Department of Medicine, Weill Cornell Medicine, New York, NY 10065, United States; NewYork-Presbyterian Hospital, New York, NY 10065, United States; Department of Population Health Sciences, Weill Cornell Medicine, New York, NY 10065, United States; Department of Psychiatry, Weill Cornell Medicine, New York, NY 10065, United States; Department of Population Health Sciences, Weill Cornell Medicine, New York, NY 10065, United States; NewYork-Presbyterian Hospital, New York, NY 10065, United States

**Keywords:** machine learning implementation, postpartum depression, electronic health record, health equity

## Abstract

**Objective:**

We developed and externally validated a machine-learning model to predict postpartum depression (PPD) using data from electronic health records (EHRs). Effort is under way to implement the PPD prediction model within the EHR system for clinical decision support. We describe the pre-implementation evaluation process that considered model performance, fairness, and clinical appropriateness.

**Materials and Methods:**

We used EHR data from an academic medical center (AMC) and a clinical research network database from 2014 to 2020 to evaluate the predictive performance and net benefit of the PPD risk model. We used area under the curve and sensitivity as predictive performance and conducted a decision curve analysis. In assessing model fairness, we employed metrics such as disparate impact, equal opportunity, and predictive parity with the White race being the privileged value. The model was also reviewed by multidisciplinary experts for clinical appropriateness. Lastly, we debiased the model by comparing 5 different debiasing approaches of fairness through blindness and reweighing.

**Results:**

We determined the classification threshold through a performance evaluation that prioritized sensitivity and decision curve analysis. The baseline PPD model exhibited some unfairness in the AMC data but had a fair performance in the clinical research network data. We revised the model by fairness through blindness, a debiasing approach that yielded the best overall performance and fairness, while considering clinical appropriateness suggested by the expert reviewers.

**Discussion and Conclusion:**

The findings emphasize the need for a thorough evaluation of intervention-specific models, considering predictive performance, fairness, and appropriateness before clinical implementation.

## Introduction

Postpartum depression (PPD) is a potentially life-threatening mental health condition with adverse maternal and infant health outcomes.^1^ It is estimated to affect 14%-20% of birthing parents in the United States.^2^ Yet, underdiagnosis and undertreatment of PPD occur in over half of the cases, making PPD prevention before symptoms arise a crucial task.^3^^,^^4^ Targets in PPD prevention include risk detection and intervention by clinicians, and social support for patients.^5^ While PPD can be screened by administering tools such as the Edinburgh Postnatal Depression Scale (EPDS), screening is not universally performed due to limited clinical resources and lack of awareness of PPD by non-psychiatric care providers.^6^ Moreover, it is known that current screening tools fail to detect risk early or individuals who conceal symptoms due to stigma or shame.^7^ Even when PPD risks are identified, the lack of individualized interventions hampers timely interventions and impedes access to appropriate care. Because of these systemic gaps, patients are often marginalized within the healthcare system, resorting to self-management without proper clinical guidance.^5^

Recent reviews of PPD prevention studies show that targeting at-risk patients based on data on risk factors may improve prevention effectiveness, and suggest digital solutions as promising platform.^3–8^ Given the significance of early detection of PPD, there is a potential to use machine learning techniques to predict PPD to facilitate timely intervention initiation. In a prior study, Zhang and Wang et al developed a machine learning model to help identify high-risk patients for PPD, utilizing electronic health records (EHRs) data from an urban academic medical center (AMC) in 2015-2018.^9^ The machine learning model is a logistic regression with L2 regularization to predict PPD. It comprised 32 features associated with mental health history, medical comorbidity, obstetric complications, medication prescription orders, and patient demographic characteristics. In a previously published retrospective study at the AMC, the performance of the model reached an area under the curve (AUC) of 0.94, precision of 0.59, and sensitivity of 0.83.

Current effort is under way to incorporate this model as clinical decision support to intervene at-risk patients in the AMC. The intervention will flag patients with risk of PPD and suggest to clinicians several approaches as preventive measure with minimal harm, including patient education, social work referrals, and lifestyle counseling. Before integration with clinical practice, a health system review process identified 2 gaps for the clinical use.^10^ The first gap is concerned with the need for clinicians to make well-informed decisions about adopting interventions, carefully determining the circumstances in which utilizing a model for clinical decision-making would be advantageous. Machine learning models require a thorough testing to establish an appropriate decision-making threshold. Failure to establish such a threshold may result in either overtreating or undertreating patients, potentially increasing the risk of unnecessary harm and undermining the potential benefits that could have been achieved. The second gap is the racial-ethnic differences in mental health services postpartum.^11^ It is well established that algorithmic bias^10^ could inadvertently perpetuate existing biases, leading to unequal access to opportunities and resources, and resulting in unfair treatment of individuals or specific groups through incorrect predictions, classifications, or recommendations. Therefore, it is crucial to investigate whether this model, which considers non-clinical features, including race, will demonstrate equivalent performance across diverse populations. This consideration has particular importance since the machine learning model interfaces with patient care in health systems, which are bound by regulatory obligations to uphold quality of care, medical ethics, and community benefit standards.

This study differs from previous work in machine learning bias in healthcare in that it is focused on a machine learning model’s implementation preparedness. We rigorously assessed the PPD prediction model’s bias, while also considering the net benefit and predictive performance—critical criteria for translating machine learning into clinical decision support. In addition, since we are implementing in patient care, we also considered the perspectives of the health systems. Thus, this study included expert reviews of the predictive model from a health system’s governance body. Considering the end-user perspective, we focused on both the quantitative metrics and the interpretability and actionability of the predictors to ensure the seamless integration of machine learning into the clinical workflow.

## Methods

### Relevant work

To examine the first gap raised in the health system review, we referred to the decision curve analysis by Vickers and Elkin,^12^ which has since found applications across various clinical fields.^13^ Decision curve analysis is a predictive model evaluation method that considers the clinical utility of decisions on whether and when to use a predictive model.^12^ At the core of decision curve analysis lies the concept of net benefit, which is a quantitative measure on the comparison between expected benefits and expected harm. By comparing net benefits across various decision thresholds and different models, decision curve analysis provides insights into the clinical utility of different decisions based on a model’s predictions in comparison to other strategies.

To address the second gap, a rigorous evaluation of algorithmic bias and fairness is crucial to ensure the safe and equitable integration of machine learning in patient care. Algorithmic bias commonly refers to disparities observed in the model outcomes with respect to certain demographic features, such as gender, race, and ethnicity.^14^ Previous literature has formalized a number of fairness definitions to assess bias and fairness.^15^ One view of fairness focuses on the predicted outcome for various subject groups. Statistical parity, in particular, requires the proportion of positive predictions to be the same across all subgroups within the target population. However, this view does not account for naturally occurring difference in outcome distribution across subgroups. Another category is based on both predicted and *actual* outcomes, which includes various metrics requiring error rates to be equal among all groups. For example, equal opportunity requires both privileged and unprivileged groups to have the similar true positive rates (TPRs) mathematically. We list a list of fairness measures from both views in Table 1.

**Table 1.** Fairness terminology and assessment measurement in research.

Concept	Definition	Metric	Detail/Calculation	Fairness range	Priority
Protected attribute	A binary grouping variable by which we want to establish fairness	N/A	Race (binary)	N/A	
Privileged value	An indicator at advantage among groups defined by the protect attribute	N/A	White race	N/A	
Statistical parity	Both privileged and unprivileged groups have equal probability of receiving positive prediction	Statistical parity difference	The difference of the proportions of positive prediction received by the non-White group to the White group (non-White—White)	[−0.1, 0.1]	Equal positive prediction across groups
		Disparate impact	The ratio of proportions of positive prediction for the non-White group to that of the White group (non-White/White)	[0.8, 1.25]	
Equal opportunity	Both privileged and unprivileged groups have equal probability of a subject in a positive class to have a negative prediction	Equal opportunity difference	The difference of true positive rates between non-White and White groups (non-White—White)	[−0.1, 0.1]	Equal error rates
Equalized odds	Both privileged and unprivileged groups have equal true positive rate and false positive rate	Average odds difference	Average difference of false positive rates and true positive rates between non-White and White groups (non-White—White)	[−0.1, 0.1]	
Predictive parity	Both privileged and unprivileged groups have equal probability of a subject with positive prediction to truly belong to the positive class	Predictive parity difference	The difference of positive predictive values between non-White and White groups (non-White—White)	[−0.1, 0.1]	Equal accuracy of positive prediction across groups

In the case of bias, previous work has proposed and reviewed various approaches to mitigate such bias in machine learning algorithms.^16–20^ Among the bias mitigation techniques, pre-processing has been found to be the most flexible and independent of the modeling algorithm. The pre-processing approach modifies the training dataset before model training. Kamiran and Calders^18^ presented 3 methods to debias, including changing the class of data subjects after ranking, assigning weights to data subjects based on subject’s expected probability with its privileged value and class divided by its observed probability (reweighing), and sampling the subjects with replacement according to their weights. Feldman et al^17^ introduced the removal of disparate impact by editing values through repair procedure and use it as features to increase fairness between groups. Calmon et al^16^ introduced a novel probabilistic formulation of data pre-processing by learning a probabilistic transformation that can modify the features and the labels in the training data. Lastly, another strategy is fairness through blindness,^21^ which excludes sensitive attributes related to unfairness, such as race or gender, from the model’s input. This approach aims to reduce bias by making the model unaware of the attributes that could potentially lead to bias. This approach is relatively easy to understand and operate, and has gained acceptance by clinical audience through studies such as Vyas et al.^22^

Among existing work on debiasing, particularly focused on medical applications are Park et al on PPD,^14^ Li et al on cardiovascular disease,^23^ Hong et al on stroke,^24^ and Thompson et al on opioid misuse.^25^ Park et al on PPD prediction evaluated multiple debiasing methods including fairness through blindness and reweighing, and found reweighing had superior to be most effective.^14^ Our work is similar to Thompson et al in that we prioritized sensitivity to not miss at-risk patients due to disparity. However, we take a comprehensive approach to debiasing to ensure the model is examined using approaches found successful from existing work while considering the health system review feedback. White not all fairness metrics can be satisfied simultaneously, this approach allows us to examine the model by capturing different views of fairness.

### Data and study population

**Figure 1.**
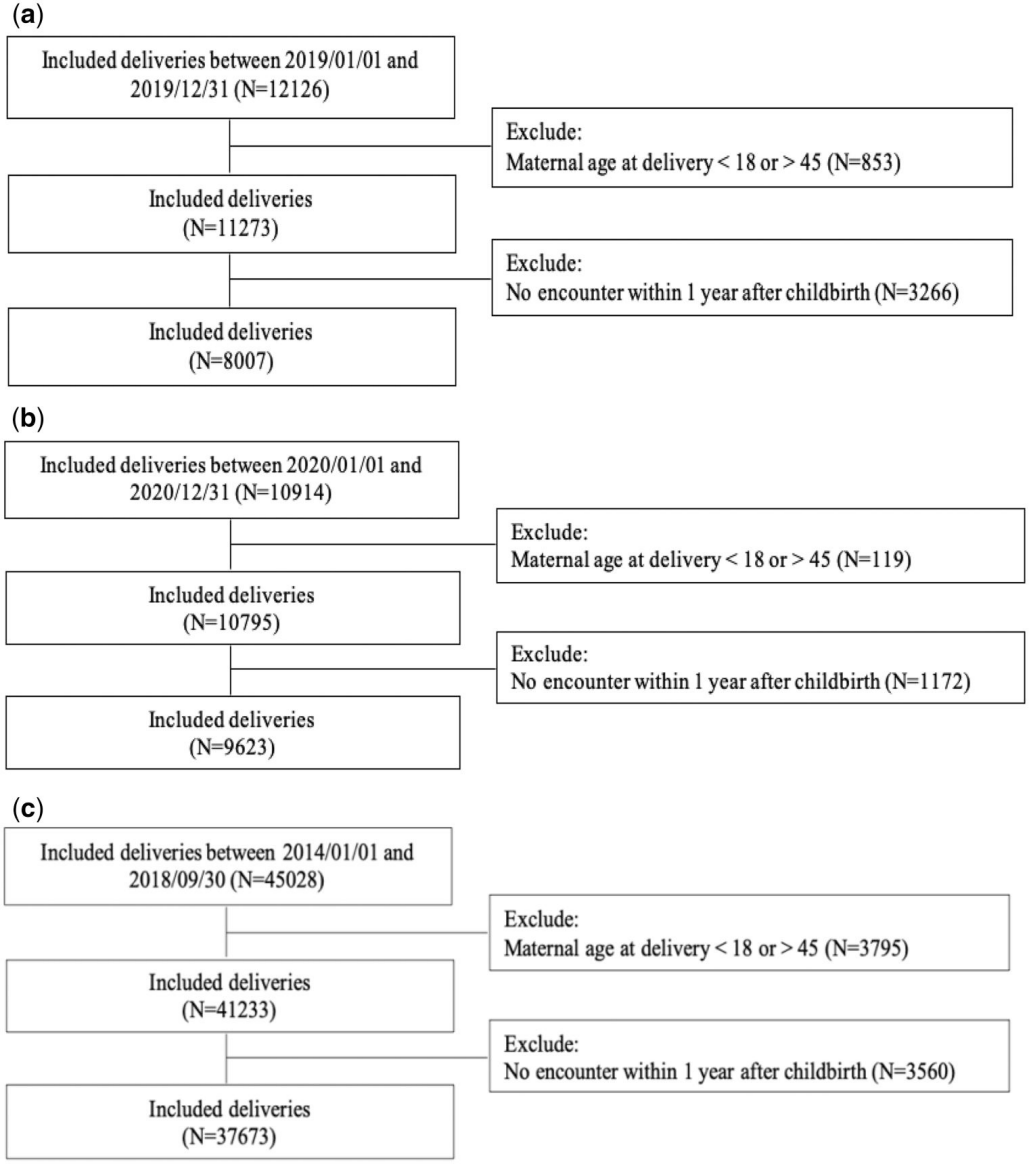
Inclusion and exclusion criteria. (a) 2019 AMC, (b) 2020 AMC, (c) Clinical research network.

To test broader generalizability across health systems and populations, we used 3 sets of EHR data separately: (1) EHR data from an AMC in 2019 (2019 AMC) which is the same site as the original development but the data is from 1 year post development; (2) to further test the model robustness, EHR data from the same AMC in 2020 (2020 AMC) which is the year of Coronavirus disease 2019 (COVID-19) pandemic; and (3) EHR data from a clinical research network between January 2014 and September 2018 (clinical research network). As shown in Figure 1, for each dataset, using the same inclusion and exclusion criteria of study population when developing PPD model,^9^ we included patients aged 18-45 who had live deliveries. The time point of prediction evaluated in this study is at childbirth. Thus, for all 3 datasets, we used data available from the beginning of pregnancy to childbirth.

### Study variables and outcome definition

Study variables used include patients’ demographics information (age, gender, race, and marital status), laboratory test results, medications, diagnoses, and mental health history. In particular, as the PPD model includes race variables, in AMC datasets, we categorized race into 5 groups: White (White, Ashkenazi Jewish, Sephardic Jewish), Asian (Asian, Asian Indian), Black (Black or African American), Other (Other Combinations Not Described, American Indian or Alaska Nation, Native Hawaiian/Other Pacific Islander), and Unknown (Declined, NA). In the clinical research network dataset, we categorized race into 5 groups: White (White), Asian (Asian), Black (Black), Other (Other, American Indian or Alaska Native, Native Hawaiian or Other Pacific Islander, Multiple race), and Unknown (Unknown, Refuse to answer, No information).

AMC EHR datasets are represented using Observational Medical Outcomes Partnership (OMOP) common data model. They include data on demographics, clinical visits, diagnoses, medications, laboratory measurements, and procedures. The clinical research network dataset is represented using National Patient-Centered Clinical Research Network (PCORnet) common data model, and include demographics, clinical visits, diagnoses, medications, and vitals. Diagnoses and medications are represented as International Classification of Diseases (ICD-9/10) codes and RxNorm Concept Unique Identifier (RxCUI)^26^ codes. To test the model in the clinical research network database, we converted the model input from SNOMED for diagnosis to ICD, and Anatomical Therapeutic Chemical for medication to RxCUI. The outcome is defined by ICD codes and regular expression applied to clinician notes. More details on the model input and the outcome definition can be found in the previous study.^9^

### Decision curve analysis

As the key factor of decision curve analysis, we calculated and compared the net benefits of different models’ clinical utility to compare the machine learning models. Term “net benefit” measures the difference between expected benefit and expected harm^13^^,^^27^ at each probability threshold. The model or threshold with higher value of net benefit is considered superior. Term “expected benefit” measures the number of patients who have the disease and who will receive the treatment using the proposed strategy. Term “expected harm” measures the number of patients without the disease who would be treated in error multiplied by a weighting factor based on patient’s threshold probability. Probability threshold is the level of diagnostic certainty above which clinicians would choose treat patients. Mathematically, at each threshold, net benefit is defined as^12^:

$$\mathrm{net}\;\mathrm{benefit}=\mathrm{expected}\;\mathrm{benefit}-\mathrm{expected}\;\mathrm{harm}=\frac{\mathrm{true}\;\mathrm{positive}\;\mathrm{count}}{n}\;–\;\frac{\mathrm{false}\;\mathrm{positive}\;\mathrm{count}}{n}\frac{P}{1-P},$$

where n is population size, false positive count = predicted positive count − true positive count, and P is the probability threshold.

Decision curve plots shows the trend of value of net benefits by probability threshold. We set the risk threshold of the model below 0.5 to enable a broader capture of potential risks, assuming that there is minimal harm and tolerable cost associated with subsequent intervention based on the model’s output.

### Fairness evaluation

The fairness definitions and metrics we used to assess our machine learning model are summarized in Table 1. We used algorithmic bias, which is mathematically defined, in modeling to measure how a model could perform differently for distinct groups. Patients having privileged value concerning the protected attribute are in the privileged group, and patients not having privileged value are in the unprivileged group. We consider race to be the protected attribute in this study and assigned White as the privileged value as opposed to Non-White. This determination draws from existing evidence on the disparity in PPD care for patients of color as known from the literature,^11^^,^^28^ where it is reported that women of color often face a heightened barrier to PPD care in comparison to their White counterparts.

Statistical parity focuses on ensuring equal prediction rates for various demographics distributions of subjects. Meanwhile, equal opportunity, equalized odds, and predictive parity largely focus on equal error rates. Equal opportunity, equalized odds, and predictive parity examine TPR, and TPR and false positive rate (FPR), and positive predictive value (PPV), respectively, while statistical parity observes the difference and ratio of positive predictions. For metrics in terms of difference, 0 indicates fairness; for metrics in terms of ratio, 1 indicates fairness. Consistent with ranges set by other research studies,^17^^,^^29–31^ the fairness ranges of the above metrics are [−0.1, 0.1], [0.8, 1.25], [−0.1, 0.1], [−0.1, 0.1], and [−0.1, 0.1], respectively.

Beyond quantitative evaluations, we underwent a review process by a health system where the AMC is campus. The health system has a governance process to review predictive models to be implemented in its EHR systems. Comprising members from the study site AMC and other AMC in the health system, informatics, and regulatory/legal fields, this governance process reviews whether predictive models have undergone peer review, external validation, and assessment for bias and fairness. In particular, the assessment of fairness and bias examines whether the model includes any sensitive variables and, if so, determines whether their inclusion was well-justified based on evidence. The review of this model included an examination of race as a predictor in the model from a modeling and end-user perspective, and questioned the use of race for its potential impact on model bias. Members in the review considered that while there is no direct link that race causes PPD, racial disparity in PPD care should be noted in the predictive modeling.^11^^,^^28^ In addition, given the workflow will include presenting clinicians with top predictors accounting for the prediction, concerns were raised on whether clinicians, who are the end-users of the clinical decision support tool based on the model, can accurately interpret and act on race as a predictor.

### Bias mitigation

Following the quantitative fairness evaluation and expert review, we undertook 5 approaches for bias mitigation (Table 2) and evaluated the impact of having race as a predictor on model performance. For the first approach, based on fairness through blindness,^21^ we used the parameters of the original logistic regression model and nullified the race-related variables with an input of 0. For the second approach, we re-trained the logistic regression model without race variables. We used reweighing in the third, fourth, and fifth approach with different nuances.^18^^,^^32^ In all reweighing approaches, reweighing was conducted by calculating weights suggested by Kamiran and Calder,^18^^,^^32^ based on subject’s expected probability with its privileged value (White race) and class divided by its observed probability. Approaches 3 and 4 removed race and then trained the logistic regression model. The weight for patients with White/Non-White race and PPD Presence/Absence is calculated as expected probability of being White/Non-White and PPD Presence/Absence divided by the actual probability of being White/Non-White and PPD Presence/Absence. For example, the weight for White and PPD patients equals to expected probability of having White and PPD divided by the actual probability of having White and PPD. Since the rates of PPD at the AMC (see Table 3) likely is the treatment prevalence rather than the actual prevalence, the fourth differs from the third approach by calculating weights based on the probability as reported in the literature, 1 in 7.^33^ For the fifth approach, we removed the race as a variable and trained the logistic regression model applying reweighing using the weights based on the expected probability of 1/7 described in the fourth approach above.

**Table 2.** Approach explanation for bias mitigation.

Approach	Definition	Race as a variable	Model parameter	PPD rates used for reweighing	*N* variables
Baseline	N/A	Yes	N/A	N/A	32
Approach 1	Fairness through blindness (F)	Nullified	Original	N/A	32
Approach 2		Removed	Retrained	N/A	30
Approach 3	Reweighing (R)	White/non-White		Observed	31
Approach 4				Literature	31
Approach 5	F and R	Removed		Literature	30

**Table 3.** Characteristics of datasets.

	2019 AMC	2020 AMC	Clinical research network
*N*	8007	9623	37673
*N* PPD (%)	788 (9.8%)	1403 (14.6%)	4336 (11.5%)
**Demographics**			
Age Mean (SD)	33.3 (4.8)	32.9 (5.3)	32.1 (5.7)
Race			
White	3557 (44.4%)	4006 (41.6%)	12301 (32.7%)
Asian	1747 (21.8%)	1190 (12.4%)	2824 (7.5%)
Black	555 (6.9%)	872 (9.1%)	5168 (13.7%)
Other	1281 (16.0%)	2140 (22.2%)	7784 (20.7%)
Unknown	867 (10.8%)	1415 (14.7%)	9596 (25.5%)
**Observed PPD prevalence rate between Non-White and White groups**			
Rate difference	−0.073	−0.067	0.001
Rate ratio	0.476	0.637	1.005

Abbreviation: AMC = academic medical center.

Results of 5 debiasing methods and original model were evaluated through a combination of 3 aspects: (1) performance including AUC, sensitivity, and precision; (2) decision curve analysis including net benefits and decision curve plots; and (3) fairness metrics including statistical parity difference, disparate impact, equal opportunity difference, average odds difference, and predictivity parity difference.

## Results

Table 3 describes the study data. In the 2019 AMC data, 788 (9.8%) deliveries were diagnosed with PPD. The mean age was 33.3 years, and 3557 (44.4%) were White. In 2020 AMC, the observed prevalence of PPD was 1403 (14.6%). The mean age was 32.9 (5.3) years, and 4006 (41.6%) were White. Compared to 2019 AMC, the observed PPD prevalence in 2020 AMC increased from 9.8% to 14.6%, potentially due to the additional burden from Covid-19 in 2020.^34^ In the clinical research network dataset, 4336 (11.5%) were diagnosed with PPD. Among them, the mean age was 32.1 years, and 12 301 (32.7%) were White. To understand the observed disease distribution, we calculated PPD prevalence rates for both Non-White and White groups, and used the difference in the prevalence rates (Non-White/White) to benchmark with statistical parity difference. Rate difference is defined as the difference in rates of PPD between the 2 groups. Similarly, we generated the ratio of the prevalence rates (Non-White/White) to benchmark with disparate impact.^35^ Rate ratio is defined as the ratio of PPD in one group to the rate of PPD in another group. The rate ratios in PPD prevalence between Non-White and White groups were 0.476 and 0.637 in the 2 AMC datasets in 2019 and 2020, respectively, and 1.005 in the clinical research network dataset. Compared to the clinical research network dataset, the rate ratios in AMC datasets show that there is an inherent difference in the PPD rates between White and non-White patients.

### Baseline model evaluation

We generated decision curve plots for the 3 datasets to examine the change of net benefits by probability threshold (Figure S1). Examining the trend of decision curves, we observe that for the 3 study datasets, the values of net benefit around 0.2 threshold were below 0. Based on the trend of decision curves and the nature of the intended intervention in the study, we examined the probability threshold between 0.3 and 0.5 and calculated the net benefits. In 2019 AMC, net benefits at 0.3 (0.049) and 0.4 (0.061) were higher than that at 0.5 (0.044). Similarly, in 2020 AMC, net benefits at 0.3 (0.102) and 0.4 (0.010) were higher than that at 0.5 (0.078). The same difference existed in the clinical research network dataset (0.061, 0.063, 0.041). The small value of net benefit is due to the small positive prediction compared to the population size.

We compared thresholds 0.2, 0.3, and 0.4 for model performance and bias. The baseline model performance and fairness evaluation at threshold 0.3 can be found in Table 4, and the results at thresholds 0.2 and 0.4 can be found in Tables S1 and S2. In the context of the baseline model’s performance, we observed that while changing thresholds does not affect AUC, lower threshold has higher sensitivity and lower precision. At lower threshold 0.2, we observe 19% and 23% to be predicted positive, in comparison to higher thresholds of 0.3 and 0.4, where 13% and 18%, and 11% and 15%, respectively, are predicted to be positive. As in previous studies with assistive clinical decision support,^25^ our model aims to offer interventions that have minimal harm to at-risk patients while limiting the workload burden from false positives. A threshold of 0.3 produces positive predictions that are close to the observed prevalence at the AMC, while a threshold of 0.2 generates more positive predictions potentially allowing us to capture more at-risk patients albeit with false positive predictions.

**Table 4.** Model performance and fairness evaluation at threshold 0.3.^a^

	AUC	Precision	Sensitivity	Statistical parity difference	Disparate impact	Equal opportunity difference	Average odds difference	Predictivity parity difference
Goal, acceptable ranges				0, [−0.1, 0.1]	1, [0.8, 1.25]	0, [−0.1, 0.1]	0, [−0.1, 0.1]	0, [−0.1, 0.1]
**2019 AMC**								
Baseline	0.96	0.48	0.95	−0.175	0.403	−**0.056**	−**0.066**	**0.051**
Approach 1	0.96	0.54↑	0.96↑	−**0.084**↑	0.619↑	−**0.009**↑	−**0.013**↑	−0.145
Approach 2	0.97↑	0.70↑	0.94	−**0.094**↑	0.49↑	−**0.045**↑	**0.017**↑	−**0.054**
Approach 3	0.96	0.75↑	0.66	−**0.062**↑	0.49↑	−**0.056**	**0.045**↑	−**0.086**
Approach 4	0.96	0.71↑	0.71	−**0.068**↑	0.499↑	−**0.03**↑	**0.012**↑	−**0.063**
Approach 5	0.97↑	0.70↑	0.72	−**0.075**↑	0.471↑	−**0.046**↑	**0.022**↑	−**0.038**
**2020 AMC**								
Baseline	0.97	0.61	0.97	−0.122	0.599	**0.005**	−**0.082**	**0.04**
Approach 1	0.96	0.66↑	0.97	−**0.048**↑	**0.805**↑	**0.028**↑	−**0.019**↑	−0.135
Approach 2	0.96	0.76↑	0.94	−**0.056**↑	0.735↑	**0.043**↑	−**0.047**↑	−**0.074**
Approach 3	0.96	0.81↑	0.66	−**0.031**↑	0.774↑	**0.03**↑	−**0.022**↑	−0.121
Approach 4	0.96	0.77↑	0.72	−**0.032**↑	0.795↑	**0.066**↑	−**0.061**↑	−**0.1**
Approach 5	0.96	0.77↑	0.73	−**0.041**↑	0.749↑	**0.037**↑	−**0.037**↑	−**0.085**
**Clinical research network**								
Baseline	0.94	0.49	0.95	−**0.011**	**0.931**	−**0.068**	**0.065**	−**0.004**
Approach 1	0.95↑	0.51↑	0.95	**0.004**↑	**1.025**↑	**0.019**↑	−**0.018**↑	**0.003**↑
Approach 2	0.95↑	0.60↑	0.94	**0.004**↑	**1.027**↑	**0.02**↑	−**0.019**↑	**0.001**↑
Approach 3	0.95↑	0.67↑	0.64	**0.024**	1.345	**0.089**	−**0.075**	−**0.08**
Approach 4	0.95↑	0.63↑	0.71	**0.008**↑	**1.083**↑	**0.05**↑	−**0.047**↑	**0.005**
Approach 5	0.95↑	0.63↑	0.71	**0.002**↑	**1.014**↑	**0.02**↑	−**0.02**↑	**0.012**

a Bolded numbers are metrics within the satisfactory fairness ranges. ↑ indicate *improvement* from baseline. Baseline: original logistic regression model. Approach 1: nullified race and use the parameters of original model. Approach 2: remove race and retrain the model. Approach 3: reweigh by White race, remove race, and retrain the model by empirical rate. Approach 4: reweigh by White according to prevalence in the literature, remove race, and retrain the model. Approach 5: remove race, retrain the model with reweighing using literature rate.

For fairness evaluation, taking the White race as the privileged value, we conducted a bias assessment comparing White and Non-White groups. The clinical research network dataset’s observed metrics are all within the defined fairness range. In contrast, in 2019 and 2020 for the AMC dataset, as shown in Table 4 and Tables S1 and S2, the metrics of equal opportunity difference, average odds difference, and predictive parity difference were largely within the accepted fairness range, indicating that there is equality in error rates. However, both statistical parity difference and disparate impact did not meet the fairness criteria. This indicates inequality in the positive prediction between Non-White group and White groups.

### Model performance and fairness evaluation for 5 bias mitigation methods

For each debiasing approach (Table 2), we evaluated the results of fairness metrics and model performance. Results at threshold 0.3 can be found in Table 4, and results at threshold 0.2 and 0.4 can be found in Tables S1 and S2. In addition, we evaluated positive predictions at thresholds 0.2, 0.3, and 0.4 in years 2019 and 2020 based on the model from Approach 2 (Table S3). Table S4 reports the model performance across White and non-White patients at threshold 0.3 across approaches. All approaches largely reached improvement in fairness metrics except for predictive parity difference. Of the 5 approaches, Approaches 2, 4, and 5 are more successful in meeting fairness than Approaches 1 and 3. For example, the fairness through blindness approach showed improvement in all metrics compared to baseline results, with statistical parity difference across all datasets increasing from outside the range to within range (from −0.175, −0.011, −0.122 to −0.094, −0.056, 0.004). while declining from baseline, predictive parity differences are still within ranges in all 3 datasets.

**Figure 2.**
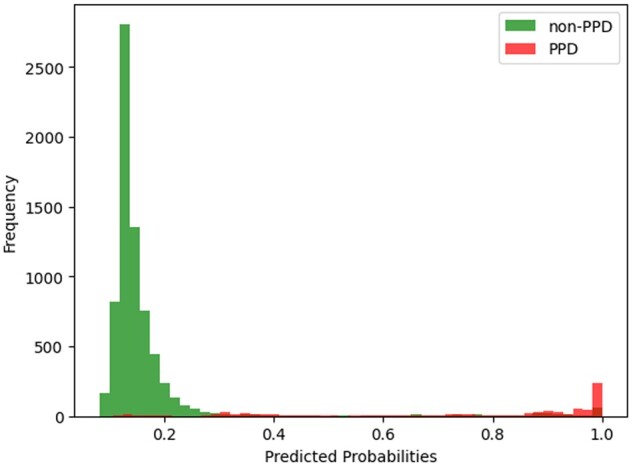
The distribution of positive and negative prediction in 2019 AMC data.

**Figure 3.**
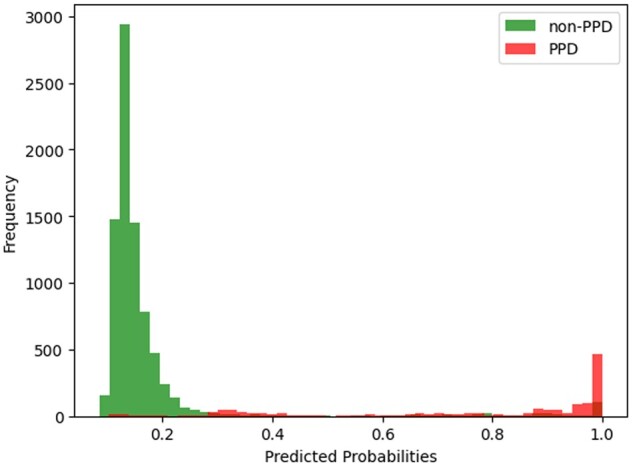
The distribution of positive and negative prediction in 2020 AMC data.

Approaches 1 and 2 had higher sensitivity and approaches 3-5 had higher precision. Compared to the first 2, our last 3 approaches had similar AUC and precision, but significantly decreased sensitivity by around 0.25. Assuming that the interventions bear minimal harm and tolerable cost, we prioritize sensitivity over precision, as the consequences of false negatives (ie, miss a patient’s risk of PPD) outweigh the consequences of false positives (ie, refer a patient with low PPD risk to patient education, social work, or lifestyle interventions). Between approaches 1 and 2 (Figure S2) we observe that the net benefit of approach 2 (fairness through blindness) at 0.3 was higher than that of approach 1. More importantly, it meets the expert feedback highlighting the difficulties clinicians might face interpreting race as a top predictor in a clinical decision support tool. Approach 2 had similar performance at thresholds 2 and 4 as shown in Tables S1 and S2. We examined the distributions of predictions in 2019 and 2020 AMC data under approach 2, as shown in Figures 2 and 3. A risk threshold of 0.3 seems to be most balanced in true positive and false positives, but a threshold of 0.2 will miss fewer at-risk patients.

This evaluation process also informed the health systems review to take a more comprehensive approach in evaluating bias in predictive models. Rather than focusing on whether a machine learning model contains sensitive attributes to determine bias, the review will place more weight on disparity in predictive performance, positive prediction, and error rates.

## Discussion

This study conducted a validation of a previously peer-reviewed and externally validated model prior to implementation in the EHR. As we prepare for machine learning’s translation to patient care, having additional examination will provide the needed transparency and justification for its output as a form of clinical decision support. Using EHR data at an AMC and a clinical research network, we assessed model fairness across cutoffs using metrics that focused on equal prediction or error. Cutoff values were determined below 0.5, based on our priority for sensitivity while minimizing potential burden from false positives in patient care. We found that the original model maintained predictive performance and, while satisfying fairness metrics that prioritized equal error rates, did not create equal positive prediction rates between White and non-White patients. We then evaluated 5 approaches to de-bias the model based on literature. Based on overall quantitative results and input from a health system review, a revised model using fairness through blindness was deemed most fit. We found that lower cutoff threshold of 0.3 for this revised model generally improved fairness. The debiasing reduced disparity as defined by equal error rates in both AMC and research network data. However, it did not reach the fairness definition of equal positive prediction rates in the AMC data. The improvement from debiasing was more salient at threshold 0.3 than at 0.2 and 0.4. We realize that we report estimates of equity based on retrospective data rather than true equity unknown from the study data alone. The debiased model’s positivity rate among patients categorized by race corresponds to the disparities in positivity seen in the AMC data. Should these differences stem from underdiagnosis in non-White patients, the positivity rates could perpetuate this disparity. Despite various methods employed to improve fairness metrics and meet the definition of fairness, the efforts achieved a limited reduction in the predicted positivity rate gap, while still maintaining sensitivity.

Our finding suggests that the results of fairness metrics are not only associated with the model but also associated with the patient characteristics and prevalence of the disease observed in the datasets. The results observed may reflect the characteristics of the populations with varying PPD rate differences and rate ratios. Compared to the AMC datasets, the clinical research network dataset’s PPD rate difference between Non-White and White groups is closer to 0. The observed PPD prevalence may explain why the model developed using data from the AMC exhibited bias as defined by statistical parity toward White patients in the AMC validation data, but was generally unbiased in a more diverse and balanced dataset of the clinical research network. This phenomenon of base rate affecting fairness has previously been discussed mathematically by Movva et al.^36^ The percentage difference in White between AMC (44.4% and 41.6%) and the clinical research network (32.7%) datasets may also account for the difference.

While it is commonly a concern that debiasing method may compromise predictive performance, for our study, they improved fairness without compromise. We recognize that fairness metrics conflict with one another and are impossible to satisfy simultaneously; the choice of the appropriate approach to mitigate bias should be context specific. We employed various methods to leverage findings from existing work and expert input. Most relevant to our study, a study by Park et al^14^ used disparate impact and equal opportunity difference to evaluate the debiasing performance for a PPD predictive model. Their study results showed the performance of reweighing was better that of fairness through blindness. Compared to their study, our study considered a combination of model performance, fairness metrics, net benefits, and end-user consideration to assess its value as a clinical decision support tool, reaching a conclusion to prefer a fairness through blindness approach with model re-training. Reasons for the difference may be the population and the implementation workflow. Park et al constructed a cohort of Medicaid enrollees in multiple states whereas our dataset came from EHR data including both commercially and publicly insured patients in an urban setting. Furthermore, our decision factored in the health system feedback that favored a fairness through blindness approach, and the balance between sensitivity and false positives. We also evaluated the debiasing approaches at 3 risk thresholds, whose different cutoff will translate to varying number of patients benefiting from the interventions.

These findings have limitations. First, our study datasets come from an urban environment from a single region of the United States. The AMC data we used have higher compositions of White patients, who have higher observed PPD prevalence than non-White patients. The clinical research network data have a more balanced racial distribution, with non-White patients having slightly higher observed PPD prevalence than White patients. Additional validations are necessary to verify our study’s broad generalizability. Secondly, the machine learning model was a logistic regression model. Future studies should evaluate implementation strategies for a more complex and black-box model. Relatedly, given that the study’s objective was not the creation of a new model, we did not evaluate the potential impact of excluding race-associated predictors on enhancing model equity. However, the removal of such predictors to improve fairness will be considered in future model development efforts. Third, this study relied on fairness metrics from the literature. Despite our best efforts, we acknowledge limitations in applying these metrics; notably, the equal error rate presumes a gold standard, while in reality, some patients needing care may be missed. Lastly, the expert review opinions are from one health system with ample resources, expertise and based on literature on machine learning fairness today. As we engage in implementation, we will monitor the model based on the fairness metrics discussed in this paper. Since PPD symptoms may emerge in various timeframes in the postpartum period, we will consider re-training when we observe statistically significant variation in predicted and expected cases, distributional shift in the data,^10^ and introduction of new clinical practices and guidelines, while being attentive to stakeholder feedback. The latest AI implementation frameworks developed for monitoring bias will also guide us in our efforts.^37^^,^^38^ As machine learning is a quickly evolving field, a larger study including health systems with low resources is needed for a national recommendation on the implementation of machine learning in healthcare.

## Conclusions

This study was motivated by the implementation of a machine learning model to predict PPD in a health system with a diverse patient population. We describe several key steps that are critical to consider, including model performance, fairness, clinical utility, and end-user consideration. Machine learning tools such as ours will be increasingly rooted in the broader context of a health system. This study aims to contribute to the literature on setting a benchmark in machine learning implementation that aligns with a mission to provide cutting-edge, equitable, and patient-centric healthcare. Our evaluation used a post-development dataset from an AMC where the initial model development took place, as well as a dataset from a clinical research network of AMCs. The findings revealed that the model exhibited biased performance in the AMC data but showed less bias in the clinical research network data, thus showing fairness metrics may vary across datasets. We evaluated 5 approaches to debias the model evaluated at 3 risk thresholds. The ultimate decision incorporated the quantitative results and expert opinions. Future work will continue to monitor the model to ensure patient outcomes and adhere to best practices in the field of machine learning.

## Supplementary Material

ocae056_Supplementary_Data

## Data Availability

The data underlying this article cannot be shared publicly due to for the privacy of individuals that participated in the study. The data will be shared on reasonable request to the corresponding author.
