# Supplementary material for: Preparing for the bedside—optimizing a postpartum depression risk prediction model for clinical implementation in a health system
Source: J Am Med Inform Assoc. 2024 Mar 26;31(6):1258–67. doi: 10.1093/jamia/ocae056 (PMC11105144; doi:10.1093/jamia/ocae056)
Supplement: ocae056_Supplementary_Data [file ocae056_supplementary_data.docx]

**eTable 1. Model performance and fairness evaluation at threshold 0.2. Bolded numbers are metrics within the satisfactory fairness ranges.** ↑ **indicate improvement from baseline.**

^0^ baseline: original logistic regression model; ^1^ approach 1: nullified race and use the parameters of original model; ^2^ approach 2: remove race and retrain the model; ^3^ approach 3: reweigh by White race, remove race, and retrain the model by empirical rate, ^4^ approach 4: reweigh by White according to prevalence in the literature, remove race, and retrain the model, ^5^ approach 5: remove race, retrain the model with reweighing using literature rate

|  | AUC | Precision | Sensitivity | Statistical parity difference | Disparate impact | Equal opportunity difference | Average odds difference | Predictivity parity difference |
| --- | --- | --- | --- | --- | --- | --- | --- | --- |
| Goal, acceptable ranges |  |  |  | 0, [-0.1, 0.1] | 1, [0.8, 1.25] | 0, [-0.1, 0.1] | 0, [-0.1, 0.1] | 0, [-0.1, 0.1] |
| **2019 AMC** | | | | | | | | |
| Baseline ^0^ | 0.96 | 0.12 | 1.0 | -0.359 | 0.641 | **-0.01** | **-0.374** | **-0.037** |
| Approach 1 ^1^ | 0.96 | 0.10 | 1.0 | **-0.003**↑ | **0.997**↑ | **0.002**↑ | **-0.003**↑ | **-0.074** |
| Approach 2 ^2^ | 0.97↑ | 0.50↑ | 0.96 | **-0.096**↑ | 0.601 | **-0.017** | **-0.019**↑ | -0.124 |
| Approach 3 ^3^ | 0.96 | 0.74↑ | 0.68 | **-0.063**↑ | 0.495 | **-0.044** | **0.031**↑ | **-0.077** |
| Approach 4 ^4^ | 0.96 | 0.70↑ | 0.75 | **-0.076**↑ | 0.485 | **-0.03** | **0.007**↑ | **-0.042** |
| Approach 5 ^5^ | 0.97↑ | 0.71↑ | 0.76 | **-0.077**↑ | 0.479 | **-0.044** | **0.021**↑ | **-0.046** |
| **2020 AMC** | | | | | | | | |
| Baseline ^0^ | 0.97 | 0.16 | 1.0 | -0.194 | **0.806** | **-0.003** | -0.217 | **-0.039** |
| Approach 1 ^1^ | 0.96 | 0.15 | 1.0 | **0.006**↑ | **1.006**↑ | **0.003** | **0.006**↑ | **-0.069** |
| Approach 2 ^2^ | 0.96 | 0.62↑ | 0.97 | **-0.058**↑ | 0.779 | **0.027** | **-0.032**↑ | -0.107 |
| Approach 3 ^3^ | 0.96 | 0.80↑ | 0.68 | **-0.035**↑ | 0.756 | **0.026** | **-0.021**↑ | -0.106 |
| Approach 4 ^4^ | 0.96 | 0.77↑ | 0.76 | **-0.041**↑ | 0.757 | **0.048** | **-0.047**↑ | **-0.085** |
| Approach 5 ^5^ | 0.96 | 0.77↑ | 0.77 | **-0.044**↑ | 0.745 | **0.034** | **-0.034**↑ | **-0.086** |
| **Clinical research network** | | | | | | | | |
| Baseline ^0^ | 0.94 | 0.40 | 0.97 | **-0.068** | **0.866** | **-0.049** | **0.042** | **-0.077** |
| Approach 1 ^1^ | 0.95↑ | 0.41 | 0.97 | **-0.049**↑ | **1.039**↑ | **0.035**↑ | **-0.037**↑ | **-0.08** |
| Approach 2 ^2^ | 0.95↑ | 0.56↑ | 0.96 | **-0.049**↑ | **1.031**↑ | **0.024**↑ | **-0.025**↑ | **-0.082** |
| Approach 3 ^3^ | 0.95↑ | 0.66↑ | 0.71 | **-0.033**↑ | **1.051**↑ | **0.023**↑ | **-0.017**↑ | -0.106 |
| Approach 4 ^4^ | 0.95↑ | 0.50↑ | 0.80 | **-0.034**↑ | **1.065**↑ | **0.032**↑ | **-0.026**↑ | -0.108 |
| Approach 5 ^5^ | 0.95↑ | 0.50↑ | 0.80 | **-0.038**↑ | **1.04**↑ | **0.02** | **-0.017**↑ | **-0.096** |

**eTable 2. Model performance and fairness evaluation at threshold 0.4. Bolded numbers are metrics within the satisfactory fairness ranges.** ↑ **indicate improvement from baseline.**

^0^ baseline: original logistic regression model; ^1^ approach 1: remove 2 race variables (White and Asian) and use the parameters of original model; ^2^ approach 2: remove 2 race variables (White and Asian) and retrain the model; ^3^ approach 3: reweigh White variable, remove Asian variable, and retrain the model, ^4^ approach 4: reweigh White variable according to literature review, remove Asian variable, and retrain the model, ^5^ approach 5: remove the 2 race variables, retrain the model with reweighing

|  | AUC | Precision | Sensitivity | Statistical parity difference | Disparate impact | Equal opportunity difference | Average odds difference | Predictivity parity difference |
| --- | --- | --- | --- | --- | --- | --- | --- | --- |
| Goal, acceptable ranges |  |  |  | 0, [-0.1, 0.1] | 1, [0.8, 1.25] | 0, [-0.1, 0.1] | 0, [-0.1, 0.1] | 0, [-0.1, 0.1] |
| **2019 AMC** | | | | | | | | |
| Baseline ^0^ | 0.96 | 0.70 | 0.87 | **-0.1** | 0.438 | -0.119 | **0.087** | **-0.039** |
| Approach 1 ^1^ | 0.96 | 0.69 | 0.84 | **-0.085**↑ | 0.486↑ | **-0.043**↑ | **0.016**↑ | **-0.049** |
| Approach 2 ^2^ | 0.97↑ | 0.70 | 0.80 | **-0.085**↑ | 0.47↑ | **-0.061**↑ | **0.035**↑ | **-0.045** |
| Approach 3 ^3^ | 0.96 | 0.78↑ | 0.64 | **-0.062**↑ | 0.468↑ | **-0.068**↑ | **0.057**↑ | **-0.07** |
| Approach 4 ^4^ | 0.96 | 0.75↑ | 0.68 | **-0.063**↑ | 0.494↑ | **-0.042**↑ | **0.029**↑ | **-0.073** |
| Approach 5 ^5^ | 0.97↑ | 0.75↑ | 0.69 | **-0.066**↑ | 0.482↑ | **-0.048**↑ | **0.033**↑ | **-0.061** |
| **2020 AMC** | | | | | | | | |
| Baseline ^0^ | 0.97 | 0.61 | 0.97 | -0.122 | 0.599 | **0.005** | **-0.082** | **0.04** |
| Approach 1 ^1^ | 0.96 | 0.66↑ | 0.97 | **-0.048**↑ | **0.805**↑ | **0.028** | **-0.019**↑ | -0.135 |
| Approach 2 ^2^ | 0.96 | 0.76↑ | 0.94 | **-0.056**↑ | 0.735↑ | **0.043** | **-0.047**↑ | **-0.074** |
| Approach 3 ^3^ | 0.96 | 0.81↑ | 0.66 | **-0.031**↑ | 0.774↑ | **0.03** | **-0.022**↑ | -0.121 |
| Approach 4 ^4^ | 0.96 | 0.77↑ | 0.72 | **-0.032**↑ | 0.795↑ | **0.066** | **-0.061**↑ | **-0.1** |
| Approach 5 ^5^ | 0.96 | 0.77↑ | 0.73 | **-0.041**↑ | 0.749↑ | **0.037** | **-0.037**↑ | **-0.085** |
| **Clinical research network** | | | | | | | | |
| Baseline ^0^ | 0.94 | 0.63 | 0.91 | **-0.048** | **0.811** | **-0.033** | **-0.017** | **0.086** |
| Approach 1 ^1^ | 0.95↑ | 0.63 | 0.90 | **0.013**↑ | **1.063**↑ | **-0.027**↑ | **0.045** | **-0.044**↑ |
| Approach 2 ^2^ | 0.95↑ | 0.64↑ | 0.90 | **0**↑ | **0.999**↑ | **-0.021**↑ | **0.023** | **-0.01** |
| Approach 3 ^3^ | 0.95↑ | 0.68↑ | 0.70 | **0.013**↑ | **1.127**↑ | **0.061** | **-0.055** | **-0.011**↑ |
| Approach 4 ^4^ | 0.95↑ | 0.65↑ | 0.66 | **0.01**↑ | **1.079**↑ | **0.05** | **-0.047** | **0.001**↑ |
| Approach 5 ^5^ | 0.95↑ | 0.66↑ | 0.66 | **0.003**↑ | **1.026**↑ | **0.016**↑ | **-0.015**↑ | **0.002**↑ |

**eTable 3. Postive predictions at thresholds 0.2, 0.3 and 0.4**

| Data | Threshold=0.2 | Threshold=0.3 | Threshold=0.4 |
| --- | --- | --- | --- |
| 2019 AMC (N=8007) | 1498 (19%) | 1060 (13%) | 902 (11%) |
| 2020 AMC (N=9623) | 2216 (23%) | 1724 (18%) | 1476 (15%) |

**eTable 4. Performance with White and non-White patients at threshold = 0.3**

|  |  | **N** | **PPD prevalence**  **(% in N)** | **PPV** | **TPR** |
| --- | --- | --- | --- | --- | --- |
| **2019 AMC** | | | | | |
| Baseline | Non-White | 4450 | 294 (6.6%) | 0.51 | 0.912 |
|  | White | 3557 | 494 (13.9%) | 0.458 | 0.968 |
| Approach 1 | Non-White | 4450 | 294 (6.6%) | 0.461 | 0.952 |
|  | White | 3557 | 494 (13.9%) | 0.605 | 0.962 |
| Approach 2 | Non-White | 4450 | 294 (6.6%) | 0.663 | 0.908 |
|  | White | 3557 | 494 (13.9%) | 0.717 | 0.953 |
| Approach 3 | Non-White | 4450 | 294 (6.6%) | 0.7 | 0.626 |
|  | White | 3557 | 494 (13.9%) | 0.786 | 0.682 |
| Approach 4 | Non-White | 4450 | 294 (6.6%) | 0.673 | 0.687 |
|  | White | 3557 | 494 (13.9%) | 0.736 | 0.717 |
| Approach 5 | Non-White | 4450 | 294 (6.6%) | 0.676 | 0.687 |
|  | White | 3557 | 494 (13.9%) | 0.714 | 0.733 |
| **2020 AMC** | | | | | |
| Baseline | Non-White | 5617 | 662 (11.8%) | 0.628 | 0.973 |
|  | White | 4006 | 741 (18.5%) | 0.587 | 0.968 |
| Approach 1 | Non-White | 5617 | 662 (11.8%) | 0.593 | 0.988 |
|  | White | 4006 | 741 (18.5%) | 0.728 | 0.96 |
| Approach 2 | Non-White | 5617 | 662 (11.8%) | 0.727 | 0.961 |
|  | White | 4006 | 741 (18.5%) | 0.801 | 0.918 |
| Approach 3 | Non-White | 5617 | 662 (11.8%) | 0.751 | 0.674 |
|  | White | 4006 | 741 (18.5%) | 0.872 | 0.644 |
| Approach 4 | Non-White | 5617 | 662 (11.8%) | 0.724 | 0.757 |
|  | White | 4006 | 741 (18.5%) | 0.824 | 0.691 |
| Approach 5 | Non-White | 5617 | 662 (11.8%) | 0.725 | 0.754 |
|  | White | 4006 | 741 (18.5%) | 0.811 | 0.717 |
| **Clinical research network** | | | | | |
| Baseline | Non-White | 25372 | 2925 (11.5%) | 0.524 | 0.940 |
|  | White | 12301 | 1411 (11.5%) | 0.438 | 0.973 |
| Approach 1 | Non-White | 25372 | 2925 (11.5%) | 0.499 | 0.943 |
|  | White | 12301 | 1411 (11.5%) | 0.544 | 0.970 |
| Approach 2 | Non-White | 25372 | 2925 (11.5%) | 0.594 | 0.933 |
|  | White | 12301 | 1411 (11.5%) | 0.604 | 0.954 |
| Approach 3 | Non-White | 25372 | 2925 (11.5%) | 0.663 | 0.656 |
|  | White | 12301 | 1411 (11.5%) | 0.674 | 0.595 |
| Approach 4 | Non-White | 25372 | 2925 (11.5%) | 0.632 | 0.724 |
|  | White | 12301 | 1411 (11.5%) | 0.631 | 0.674 |
| Approach 5 | Non-White | 25372 | 2925 (11.5%) | 0.632 | 0.716 |
|  | White | 12301 | 1411 (11.5%) | 0.62 | 0.697 |

**eFigure 1. Baseline decision curve plots**


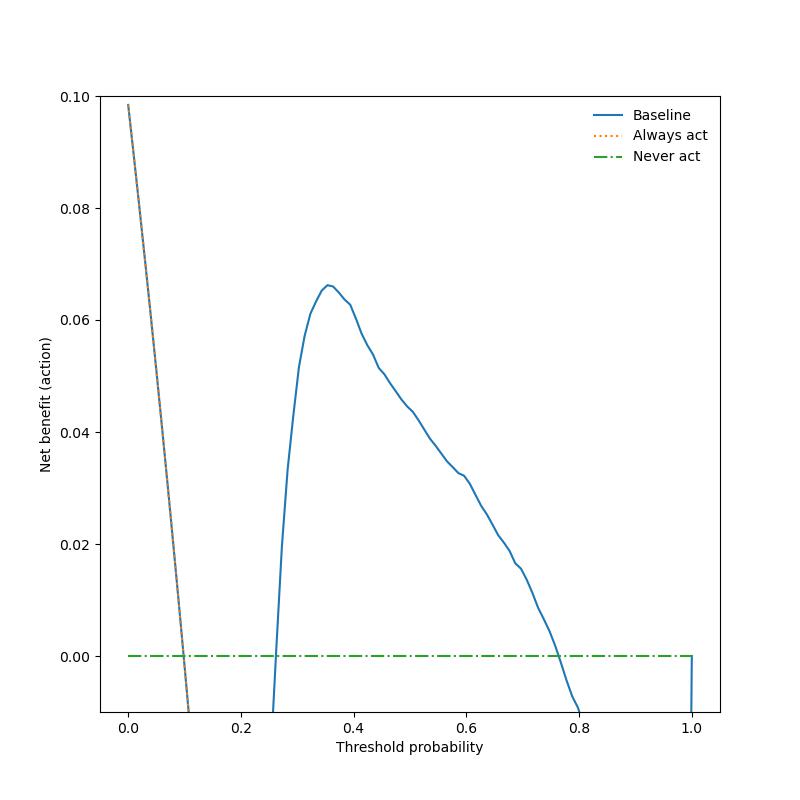

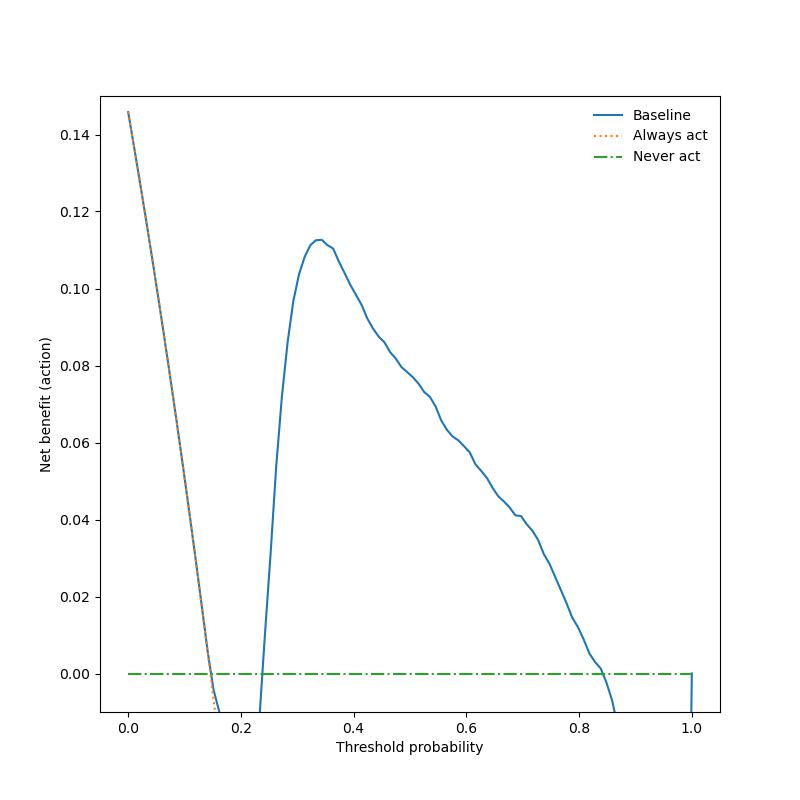


eFigure 1a 2019 AMC eFigure 1b 2020 AMC


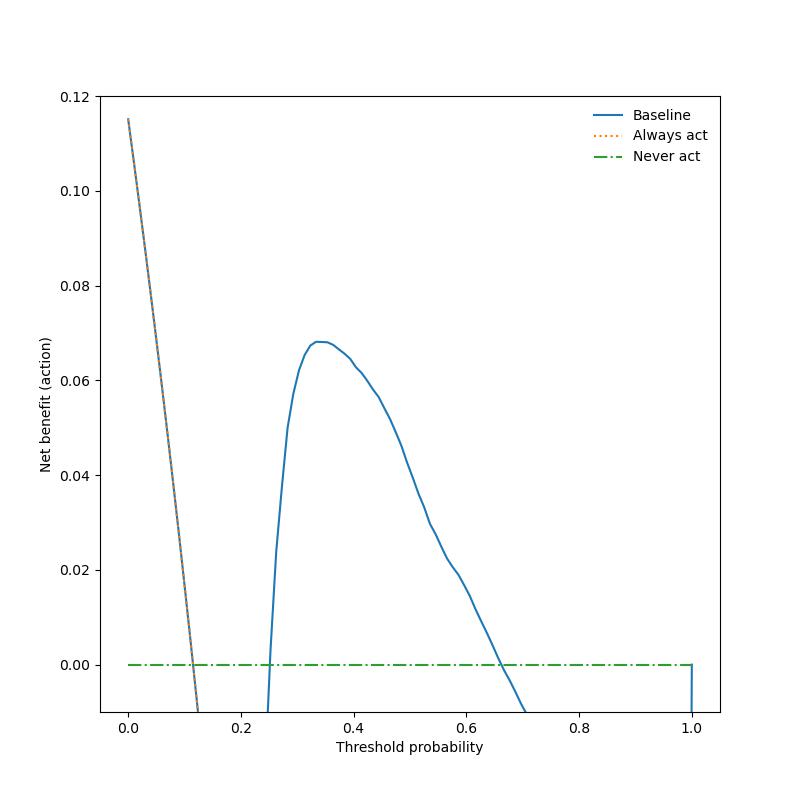


eFigure 1c Clinical research network

**eFigure 2. Decision curve plots for five bias mitigation methods**


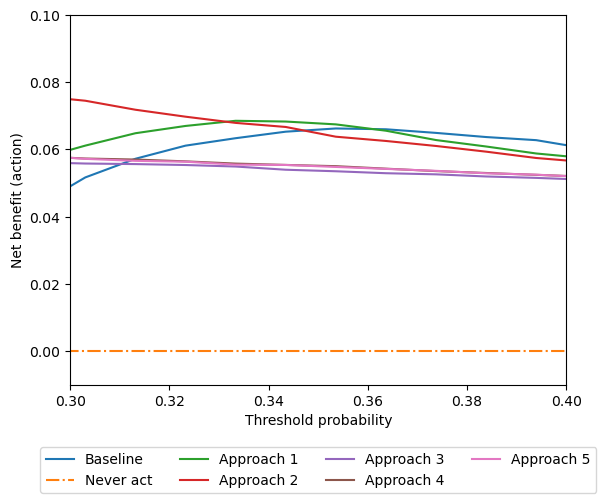

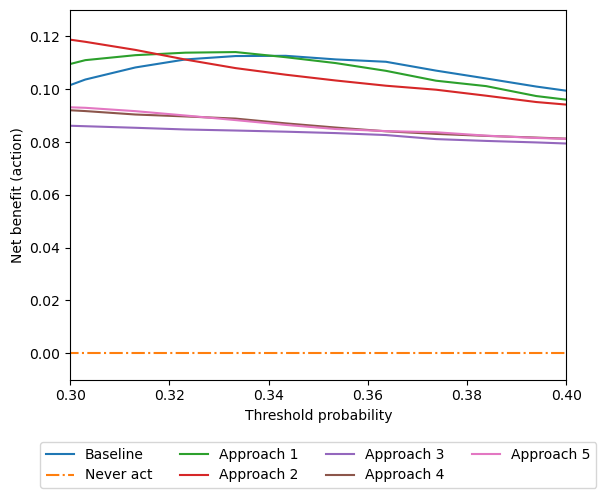


eFigure 2a 2019 AMC eFigure 2b 2020 AMC


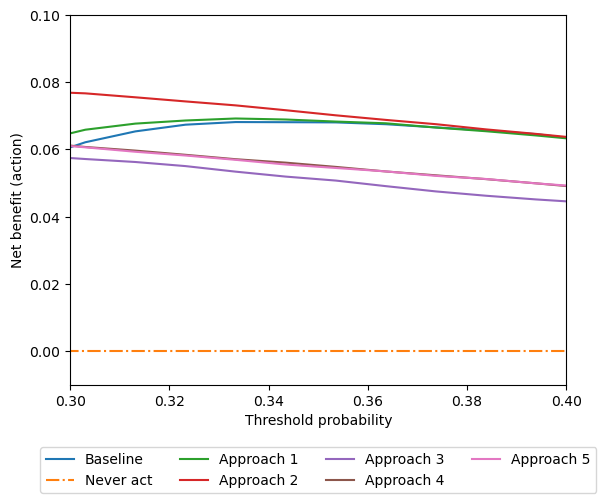


eFigure 2c Clinical research network
